# Degradation of Biofumigant Isothiocyanates and Allyl Glucosinolate in Soil and Their Effects on the Microbial Community Composition

**DOI:** 10.1371/journal.pone.0132931

**Published:** 2015-07-17

**Authors:** Franziska S. Hanschen, Bunlong Yim, Traud Winkelmann, Kornelia Smalla, Monika Schreiner

**Affiliations:** 1 Department of Quality, Leibniz-Institute of Vegetable and Ornamental Crops Grossbeeren/Erfurt e.V., Theodor-Echtermeyer-Weg 1, D-14979 Grossbeeren, Germany; 2 Institute of Horticultural Production Systems, Leibniz Universität Hannover, Section of Woody Plant and Propagation Physiology, Herrenhäuser Str. 2, D-30419 Hannover, Germany; 3 Institute for Epidemiology and Pathogen Diagnostics, Julius Kühn-Institut – Federal Research Centre for Cultivated Plants (JKI), Messeweg 11–12, D-38104 Braunschweig, Germany; Graz University of Technology (TU Graz), AUSTRIA

## Abstract

Brassicales species rich in glucosinolates are used for biofumigation, a process based on releasing enzymatically toxic isothiocyanates into the soil. These hydrolysis products are volatile and often reactive compounds. Moreover, glucosinolates can be degraded also without the presence of the hydrolytic enzyme myrosinase which might contribute to bioactive effects. Thus, in the present study the stability of *Brassicaceae* plant-derived and pure glucosinolates hydrolysis products was studied using three different soils (model biofumigation). In addition, the degradation of pure 2-propenyl glucosinolate was investigated with special regard to the formation of volatile breakdown products. Finally, the influence of pure glucosinolate degradation on the bacterial community composition was evaluated using denaturing gradient gel electrophoresis of 16S rRNA gene amplified from total community DNA. The model biofumigation study revealed that the structure of the hydrolysis products had a significant impact on their stability in the soil but not the soil type. Following the degradation of pure 2-propenyl glucosinolate in the soils, the nitrile as well as the isothiocyanate can be the main degradation products, depending on the soil type. Furthermore, the degradation was shown to be both chemically as well as biologically mediated as autoclaving reduced degradation. The nitrile was the major product of the chemical degradation and its formation increased with iron content of the soil. Additionally, the bacterial community composition was significantly affected by adding pure 2-propenyl glucosinolate, the effect being more pronounced than in treatments with myrosinase added to the glucosinolate. Therefore, glucosinolates can have a greater effect on soil bacterial community composition than their hydrolysis products.

## Introduction

Recently, plant-derived isothiocyanates (ITC) gained interest as natural ‘pesticides’ in a process termed biofumigation, that aims to reduce soil-borne pests and pathogens by incorporating plants of the Brassicales order into the soil. The reactive ITC are formed enzymatically from glucosinolates (GLS) (*β*-d-thioglucoside-*N*-hydroxysulfates), secondary plant metabolites in Brassicales members [1]. To date, more than 130 GLS that can be classified into aliphatic, aromatic, and indole GLS, have been identified in plants belonging predominantly to the Brassicaceae family [2]. These compounds are present in all organs of brassicaceous plants but their composition and concentrations differ enormously [3,4]. After tissue disruption GLS encounter myrosinase, a *β*-d-thioglucosidase that cleaves *β*-d-glucose. The resulting aglucon spontaneously degrades to form ITC, nitriles or, if an additional protein is present, epithionitriles [5]. Especially ITC show antimicrobial [6,7] and antinematicidal [8,9] activities, thereby being promising biofumigants. However, the soil microorganisms can also have positive effects for plants, such as plant growth promotion by certain bacteria [10]. Thus, GLS- and ITC-induced changes of the bacterial and fungal community composition will likely contribute to the biofumigation effects.

During the biofumigation process, GLS-rich species or materials are crushed and mixed into the soil in order to release the volatile and toxic ITC [11]. Conversion of GLS to hydrolysis products during field experiments depends not only on the GLS concentration within the plant but on the extent of tissue disruption, the soil temperature and the water content as well [12,13]. Concentrations of ITC in treated soils usually range between 1 and 100 nmol ITC/g soil [14]. Studies taking samples after several periods in time revealed that ITC concentration was highest at the first sampling [11,12,15]. After a biofumigation treatment, in some studies no ITC were detectable after three days [11], whereas others reported an increment in ITC concentrations after three days that was attributed to increased water content of the soil [12]. Concentrations of ITC can decline due to their volatility [16], due to sorption to soil particles [17,18], due to biodegradation [16,18], and probably due to chemical degradation as well [16,17]. Sorption of ITCs is based on their lipophilic character and increases with lipophilicity [18]. The process is fast and higher in soils with high organic matter content [16–18]. Thereby, concentration of ITCs in gaseous phase and its toxicity can decline [14]. With regard to biodegradation, the microbiota has been identified as a major factor for ITC reduction in soils [16]. Biodegradation, which is also known for pesticides, increases with higher pH value and if soils have been treated with the compound or chemically related compounds several times [19,20]. Chemical degradation of ITCs probably also accounts for decline in concentration, as ITCs readily react with nucleophilic compounds such as thiols, amines, or water, their reactivity increasing with temperature and pH value [1,21].

For biofumigation treatments usually crops with high GLS content are chosen such as Indian mustard (*Brassica juncea*), which is rich in 2-propenyl GLS (allyl-GLS) or 4-methylthio-3-butenyl GLS (4-MT-3But-GLS) containing oilseed radish (*Raphanus sativus* var. *oleiformis)* [12,22,23]. However, often not all the GLS are degraded by plant myrosinase and intact GLS might be released into the soil by root exudation of *Brassica* plants [24]. Yet, intact GLS can be also degraded in soil without the presence of plant myrosinase, still being able to contribute to biofumigation. Gimsing et al. studied the degradation of two aliphatic (but-3-enyl and 2-hydroxy-but-3-enyl) and two aromatic (benzyl and 2-phenylethyl) GLS in different soils. They observed that benzyl-ITC was the main degradation product after degradation of its pure GLS in soil [25], whereas the degradation was largely independent of the specific GLS-structure but affected by the soil type [26]. Extracellular non-*Brassica* derived myrosinase was made responsible for degradation, as autoclaving of soil enhanced GLS-stability enormously but sodium azide or γ-irradiation did not [26].

However, up to now it is not clear whether the GLS breakdown product profile can differ between different soils. This would strongly affect the biofumigation effect. Moreover, it has been shown that biofumigation treatments with broccoli or treatment with metham sodium can influence the microbial soil community [27], but seems to be dependent on the kind of treatment. Exemplarily, 2-phenylethyl ITC given as single, unique dose [61 nmol/g soil (sandy loam)] had no effect in that study [27]. However, in another study 2-phenylethyl ITC added subsequently in multiple doses to a luvisol soil daily for five days (1.3–4 nmol/g soil/ day) was shown to affect the soil microbial community [28]. Thus, effects on microbial communities probably are strongly dependent on the treatment and on the soil as well. With regard to plant-environment interactions it was shown that GLS and ITC can induce changes in microbe populations in the rhizosphere and on the plant roots[28,29]. Moreover, as pure GLS were already shown to affect microbial respiration rate in soil [30], also the pure GLS might affect the microbial community and could influence its composition as well as their corresponding ITC.

The intensive replanting of woody species, especially in members of the *Rosaceae* family often causes reduction of plant vigor and growth [31]. Therefore, biofumigation might be a tool to fight the so called replant disease, that especially leads in nurseries producing rootstocks or grafted plants of apple and rose to high economic losses. Thus, the present study aimed to examine selected aspects of biofumigation under model conditions, using reference soils from three different tree nurseries in northern Germany − a region typical for production of woody plants. Also, under these model conditions it was assessed how degradation of pure allyl-GLS with or without addition of plant myrosinase in the three reference soils might differ. The experiment aimed to determine exemplarily the degradation pathways of an individual GLS that is typical for biofumigation crops. Moreover, in a first approach, by using denaturing gradient gel electrophoresis (DGGE) analysis of 16S rRNA gene fragments amplified from total community DNA, it could be evaluated whether such treatments generally affect soil bacterial community. Further, as there is little information on the stability of different ITCs and nitriles in soils, the stability of GLS-breakdown products derived from typical biofumigation plants (Indian mustard and oilseed radish) was studied as well.

## Material and Methods

### Chemicals and Materials

2-propenyl glucosinolate hydrate (sinigrin, allyl-GLS, ≥ 99%), 2-propenyl isothiocyanate (allyl-ITC, ≥ 99%), benzonitrile (≥ 99.9%), 3-butenenitrile (allyl-CN, ≥ 98%), thioglucosidase (myrosinase) from *Sinapis alba* (white mustard) seed (EC 3.2.1.147), 2-phenylethyl isothiocyanate (2-phenylethyl-ITC, ≥ 99%), and 3-phenylpropanenitrile (2-phenylethyl-CN, ≥ 99%) were purchased from Sigma-Aldrich Chemie GmbH (Steinheim, Germany). 4-(Methylthio)butyl ITC (4-MTB-ITC; ≥ 98%) was purchased from Santa Cruz Biotechnology (Heidelberg, Germany); 1-cyano-2,3-epithiopropane (CETP, ≥ 95%) was purchased from Taros Chemicals GmbH & Co. KG (Dortmund, Germany); methylene chloride (GC Ultra Grade) from Carl Roth GmbH (Karlsruhe, Germany); acetonitrile (Ultra Gradient HPLC grade) from J.T. Baker (Deventer, The Netherlands); iron standard (1 g/L Fe, (FeCl_3_ in 15% HCl) Titrisol), HNO_3_ (65%, Suprapur) and H_2_O_2_ (30%, Suprapur) were purchased from Merck KGaA (Darmstadt, Germany). NaSO_4_ (≥ 99%) was purchased from VWR International GmbH (Darmstadt, Germany). Water was of Milli-Q quality.

### Soil and Plant Material

The three reference soils originating from three different tree nurseries growing Rosaceae rootstocks in the area of Pinneberg, Germany were chosen for the experiments, namely AL (53°42' 18.5" N, 9°48' 15.9" E), KL (53°41' 56.9" N, 9°40' 59.2" E) and ML (53°44' 24.5" N, 9°46' 54.0" E). The owners of the land gave permission to conduct the study on these sites. The soils were taken from the A-horizon of grass allotments where no *Brassica* crops had been grown at least during the last five years and it was confirmed that taking the soil did not endanger protected species. Characteristics of the three different soil types are given in Table 1.

**Table 1:** Reference soils used in this study.

Soil	Soil type	pH_CaCl2_	Fe (μg/g)^a^	Clay (%)	Silt (%)	Sand (%)	Organic matter (%)	N_Total_ (mg/g)	C_Total_ (mg/g)	C/N_Ratio_
**AL**	Slightly loamy sand	4.8	1.55	7.4	13.9	78.7	3.7	1.14	15.3	13.5
**KL**	Sandy soil	5.3	0.56	3.1	4.3	92.6	4.2	1.59	23.2	14.6
**ML**	Slightly loamy sand	5.3	0.56	7.0	17.3	75.6	4.6	1.41	22.7	16.1

^a^soluble iron content

Oilseed radish, *Raphanus sativus* var. *oleiformis*, cv. ‘Defender’ and Indian mustard, *Brassica juncea*, cv. ‘Terraplus‘ plants were grown in the greenhouse (Leibniz Universität Hannover, Germany) in 5 L pots for eight weeks at 20 ± 2°C, under 16 h photoperiod. Irrigation was applied on a daily basis and plant protection was carried out according to horticultural practice with mainly spraying against insect pests. After harvest, the plants were frozen at -50°C and stored for one night.

### Determination of soluble iron content of soil by flame-AAS spectroscopy

In order to determine the soluble iron in the soil, the method reported previously has been adapted [32]. Ten g of dry soil were extracted with 5 mL of 0.14 mM HNO_3_ (pH 2.8) using 10 min of ultrasonification. After a centrifugation step, the water phase was filtered through an ash-free filter paper and soils were re-extracted twice as described above. Combined extracts were dried in a shaking heater and digested with 200 μL of a mixture of hydrogen peroxide (30%) and concentrated HNO_3_ (v/v 1:1) in a shaking heater at 95°C to dryness. Prior to analysis, the residue was dissolved in 1 mL of 10 Vol. % HNO_3_ and dissolved to 0.2%. Iron was determined by flame-AAS spectroscopy using an AAS Vario 6 (Analytik Jena AG, Germany). Iron content was calculated by using standard addition procedure (0.2–1 mg/L) to the sample at a wavelength of 248.3 nm and a slit width of 0.2 nm using deuterium background correction.

### Determination of total nitrogen and total carbon in soil by elemental analysis

The content of total nitrogen and total carbon in soil was measured according to the method of DUMAS [33] using the elemental analyzer Vario EL Cube (Elementar Analysensysteme GmbH, Hanau, Germany).

### Degradation of plant-derived glucosinolates in soil: model biofumigation

In order to determine stability of different GLS breakdown products in soil, 180 g of each soil type were filled in a 200 mL glass beaker. Oilseed radish or Indian mustard material was crushed with a homogenizer HO 4 (Edmund Bühler GmbH, Hechingen, Germany) after adding water in a ratio of 1.1:1 (plant/water (w/w)). Fifty g of homogenized plant material were added to the soil (making 0.145 g plant/g soil) and thoroughly mixed with a stirring rod. Beakers were closed with a glass lid and Parafilm and stored in the dark at room temperature (22°C) until analysis. Samples were taken after 10 min, 1, 2, 6, and 24 h. Treatments were carried out in three biological replicates.

### Degradation of pure allyl-GLS and allyl-ITC in soil

For the degradation experiments of pure allyl-GLS and allyl-ITC in the different soils, soils were sieved through a 2 mm sieve. Then, the water holding capacity was set to 100% to conduct the model experiment at high water content similar to earlier studies [26,30]. 50 g of soil were weighted into a 50 mL glass vial with snap cap. Three different treatments were carried out, I: 0.32 μmol /g soil of allyl-GLS were added, II: 0.16 μmol /g soil of allyl-GLS and 1 unit of myrosinase were added, III: 0.1 μmol /g soil of allyl-ITC were added. These concentrations were chosen as they are similar to concentrations used in previous investigations and reported to be of practical relevance, too [14,25]. Concentration of the GLS was doubled in treatment I in contrast to treatment II, as it was assumed that levels of degradation products would be much lower compared to treatment II. Treatment I and III were also carried out with freshly autoclaved soil (121°C, 20 min). After adding the substances, they were quickly mixed into the soil and lids were closed until analysis. Samples for the GLS and hydrolysis product analysis were taken immediately and after 6 h (only for treatments II and III), after one day and two days (only for the experiment with autoclaved soil and treatment I), after seven and 28 days (only for the experiment with non-autoclaved soil and treatment I). Samples for DGGE analysis were taken after seven days (treatments I and II). Non-treated controls were stored in the same way as treated samples. All treatments were carried out in three (autoclaved soil) or four (non-autoclaved soil) biological replicates.

### DGGE analysis of soil bacterial communities

Seven days after treatment with pure allyl-GLS, soil total community DNA isolation was carried out from 500 mg of homogenized soil (sieved through 2 mm mesh size) applying the FastDNA SPIN Kit, and purifying by the GENECLEAN SPIN Kit for soil. Isolation and purification of the DNA followed the manufacturer’s instructions (MP Biomedicals, Heidelberg, Germany). Amplification of bacterial 16S rRNA gene fragments (GC-PCR) was carried out with 5x PCR GoTaq buffer, 0.2 μM dNTPs, 3.75 mM MgCl_2_, 4% acetamide (Promega GmbH, Mannheim, Germany), primers F984GC with GC-clamp and R1378 (0.2 μM each) [34], 1.25 U GoTaq (Promega GmbH, Mannheim, Germany), and 1 μL purified DNA extracts diluted 10 fold. The amplification was carried out at 94°C for 5 min, followed by 35 cycles of 94°C for 1 min, 53°C for 1 min and 72°C for 2 min and finally 72°C for 10 min [34].

The GC-PCR products of the bacterial 16S rRNA gene fragments were checked by agarose gel electrophoresis after ethidium bromide staining, loaded in the denaturing gradient gel electrophoresis (DGGE) gradients, and silver-stained as described previously [34–36]. The air-dried gels were scanned, and analyzed with Gel-Compar II 6.5 (Applied Math, Sint-Martens-Latern, Belgium) software.

### Determination of GLS

GLS concentrations in soil and plant samples were determined as desulfo-GLS using the DIN EN ISO 9167–1 based method described previously by Wiesner et al. [37] with slight modifications, i.e. 500 mg of the soil samples were lyophilized (or 20 mg of lyophilized plant material were used), extracted, and analyzed by UHPLC-DAD using a UHPLC Agilent 1290 Infinity system (Agilent Technologies, Böblingen, Germany) with a Poroshell 120 EC-C18 column, 100 mm x 2.1, particle size 2.7 μm (Agilent Technologies). Analytes were separated using a gradient consisting of water (A) and 40% acetonitrile (B) as follows: 2 min 0.5% B, increasing to 49.5% B within 10 min and holding this percentage for 2 min. Then the column was washed by increasing concentration of B within 1 min to 99.5% and eluting for another 2 min. Finally the column was equilibrated with 0.5% B for 2 min. Flow rate was 0.4 mL/min and injection volume was 5 μL. Desulfo-GLS were identified by comparing retention times and UV absorption spectra with those of known standards. Quantification was done by using an external calibration curve with allyl-GLS and a wavelength of 229 nm and GLS were calculated using response factors (RF) relative to allyl-GSL as reported previously [37].

### Determination of GLS hydrolysis products in soil samples

For the determination of GLS breakdown products, the method of Witzel et al. [38] was adapted. An aliquot of soil sample (1.5 g for the allyl-GLS experiment, 4 g for the model biofumigation experiment) was weighted in centrifugal tubes and extracted as described before using benzonitrile as internal standard [38]. Samples were analyzed with the gas chromatography-mass spectrometry detection (GC-MS) system reported previously [38] with slightly modified conditions. The temperature gradient was 35°C (3 min) to rise up to 50°C with 9°C/ min. After holding this temperature for 7 min, the temperature increased a) directly to 230°C with 9°C/ min and was held for 1 min (selected ion monitoring (SIM) method for the pure allyl-GLS/ITC experiment), or b) to 100°C with 9°C/ min and held for 3 min, then further increased with 3°C/ min to 223°C, with 9°C/ min to 230°C and was held for 1 min (full scan mode (TIC)), and finally both gradients increased with 35°C/ min to 310°C. The temperature of the transfer line was 310°C, the ion source of the mass selective detector was set to 230°C. Mass spectra (EI, 70 eV) were acquired in the full scan mode (TIC) for identification and quantitation of plant-derived breakdown products (*m/z* 30–350) and in SIM mode for quantitation of allyl-GLS breakdown products. The quantifier ion for allyl-CN was m/z 41, allyl-ITC was quantified using m/z 99.

Analytes were identified by comparing mass spectra and retention times with those of authentic standards and with literature data [39,40]. Analyte content was calculated using benzonitrile as internal standard and the response factor (RF) of each compound relative to benzonitrile. The RF were experimentally determined for allyl-ITC (RF_TIC_ = 1.70, RF_SIM_ = 3.07), allyl-CN (RF_TIC_ = 3.70, RF_SIM_ = 7.32), CETP (RF_TIC_ = 2.10, RF_SIM_ = 4.85), 2-phenylethyl-ITC (RF_TIC_ = 0.68), 2-phenylethyl-CN (RF_TIC_ = 0.82), and 4-MTB-ITC (RF_TIC_ = 0.76). The degradation products of 4-methylthio-3-butenyl (4-MT-3But) glucosinolate were calculated in the TIC mode with the RF of 4-MTB-ITC, as this compound is chemically most similar. The limits of detection ranged between 2 μM (4-MTB-ITC) and 8 μM (2-phenylethyl-ITC) in the TIC mode and from 0.1 μM (allyl-CN) to 0.3 μM (allyl-ITC) in the SIM mode.

### Fitting of data

Results from the degradation of pure and plant derived ITC could be fitted by a first order reaction model with [A]_t_ being the concentration at a certain time (t), [A]_0_ being the concentration at the beginning (t = 0), k being the reaction rate constant and t_1/2_ being the half-life period (time when 50% will be degraded):

[A]t=[A]0 · e−k·t (1)

t1/2=ln2 / k (2)

### Statistics

To investigate the effects of soil type, hydrolysis product structure, or time on the degradation of allyl-GLS and GLS-hydrolysis products, one-way analysis of variance (ANOVA) was performed. For the comparison of means Fischer LSD test was applied using the STATISTICA version 12 software [StatSoft, Inc. (2013)]. With regard to the DGGE-analysis, the air-dried gels were scanned, and analyzed by Gel-Compar II 6.5 (Applied Math, Sint-Martens-Latern, Belgium) using Pearson correlation and Unweighted Pairwise Grouping Method Using Arithmetic Means (UPGMA). The matrix data from the GelCompar analyses were taken to test for significant variability between groups, performing a permutation test at 10,000 times as described previously [41].

## Results

### Degradation of plant derived glucosinolate degradation products in soils

**Fig 1:**
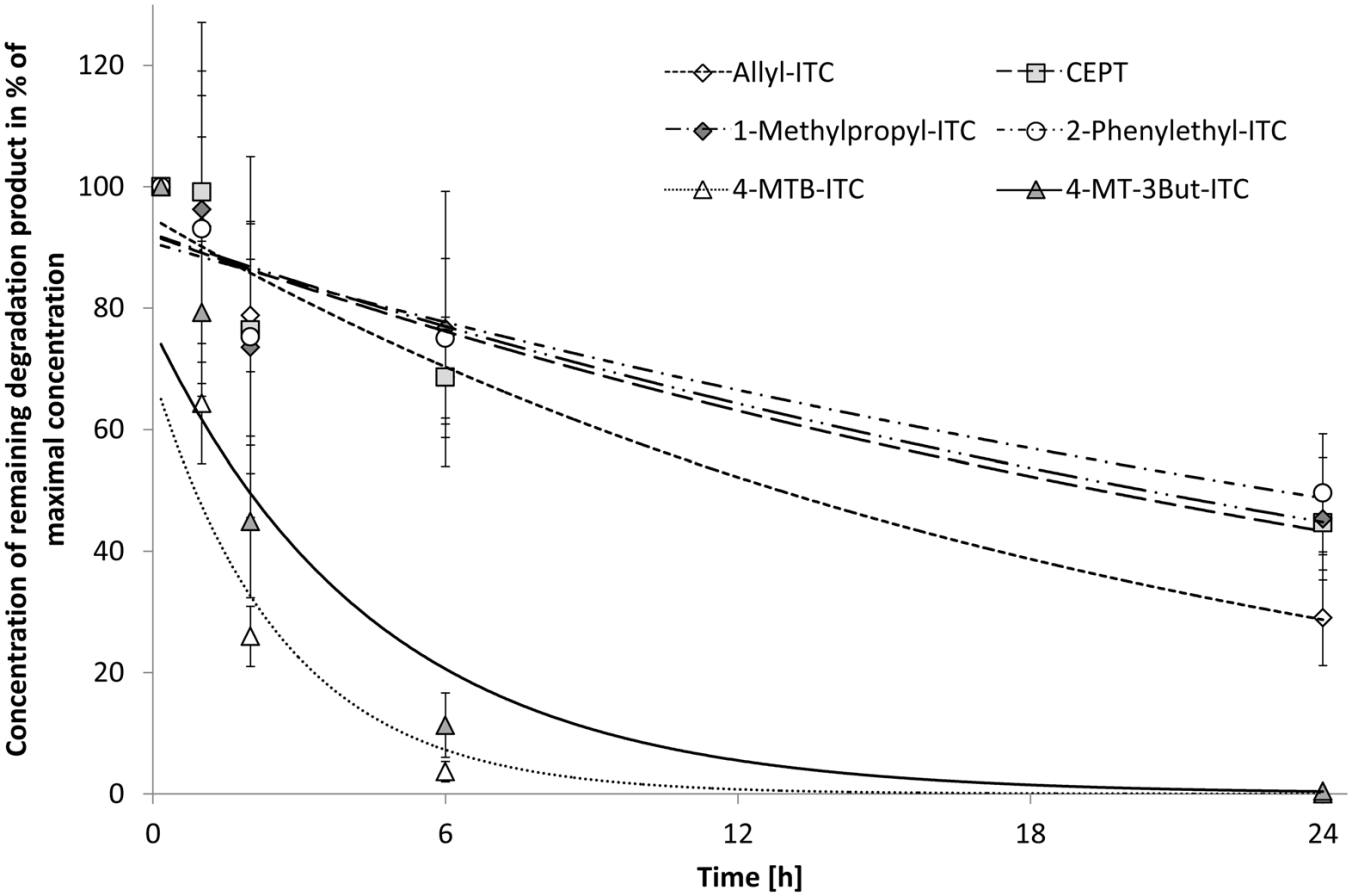
Degradation of plant-derived GLS hydrolysis products in ML soil. The lines represent the fit of the data to a first-order degradation curve. The bars show the standard deviation for three replicates.

Homogenized plant tissues of oilseed radish and Indian mustard were incorporated into three different soils in order to study the stability of the GLS hydrolysis products in these soils at room temperature. Indian mustard released mainly allyl-ITC (63%) and 2-phenylethyl-ITC (20%). Additionally, allyl-CN (5%), 1-cyano-2,3-epithiopropane (CETP) (8%), the epithionitrile deriving from allyl-GLS, 1-methylpropyl-ITC (sec-butyl-ITC) (4%), and 2-phenylethyl-CN (0.4%) have been detected. Oilseed radish formed mainly 4-methylthio-3-butenyl ITC (4-MT-3But-ITC) (97%), 4-MTB-ITC (3%) and small amounts of 5-methylthio-4-pentenyl nitrile (4-MT-3But-CN) (0.3%) upon hydrolysis. Analyzing GLS content of plants prior to and after tissue homogenization as well as formation of hydrolysis products revealed that degradation of GLS to hydrolysis products was > 99% and recovery of corresponding breakdown products ranged between 67 and 99%. Usually, after the first sampling (10 min after incorporation), the highest breakdown product concentration was detected which declined thereafter. However, in the KL soil with the mustard-treatment, the highest concentrations were observed after 1 h. Therefore, in this case for the calculation of the kinetic data the 10 min-value was excluded. The five ITC compounds and one epithionitrile were not stable in the soils but their concentrations declined rapidly within 24 h (Fig 1). The kinetic data are presented in the supporting information S1 Table. The stability of five out of these six degradation products did not differ significantly among the different soils (Fischer LSD test). Only sec-butyl-ITC concentration showed a slight difference between ML (a), AL (ab) and KL (b) using Fischer LSD test, a difference that was non-existent if a Tukey’s HSD test was applied.

Although there were no differences among the soils, however, great differences were observed between the different GLS hydrolysis products: Hydrolysis products derived from aliphatic GLS containing sulfur in their side chain [methylthioalk(en)yl hydrolysis products] in oilseed radish were much more labile (factor 20–50) than the aliphatic and aromatic ones released by the Indian mustard: 4-MTB-ITC was least stable of all GLS hydrolysis products with half-life periods of about only one hour (S1 Table and Fig 1). The chemically similar 4-MT-3But-ITC was also very labile.

Furthermore, in the experiment using Indian mustard, the nitriles allyl-CN and 2-phenylethyl-CN were formed in relatively low levels. They were relatively stable and did not decrease significantly within 24 h. In contrast, the 4-MT-3But-CN that was present in very low concentrations in oilseed radish decreased with time and was absent in most samples after 2 h and in all samples after 6 h.

### Decrement of pure allyl-ITC in soils

Degradation of pure allyl-ITC concentrations was investigatedin the three different soils. As biodegradation has been described as the main factor degrading ITC in soils [18], degradation in autoclaved soil was studied as well for the KL soil. Again, stability of allyl-ITC did not differ among the soils (see Table 2). Moreover, half-life periods were similar to the first experiment (S1 Table). Autoclaving significantly increased the ITC stability, but degradation was still observable and significant after 48 h (Table 2).

**Table 2:** Kinetic parameters of pure allyl-ITC degradation in three reference soils (AL, KL, ML) obtained by fitting of the eqs 1 and 2.

Soil	k [h^-1^]	t _1/2_ [h]	R
AL	0.052 ± 0.003	13.30 ± 0.81	0.990 ± 0.006
KL	0.049 ± 0.012	14.92 ± 3.38 (a)	0.976 ± 0.012
ML	0.051 ± 0.012	13.89 ± 1.65	0.993 ± 0.008
KL autoclaved	0.0042 ± 0.0007	168.6 ± 29.7 (a)	0.998 ± 0.006

Small letters in brackets indicate significant differences between the half-life periods of allyl-ITC at the p < 0.05 level (Tukey’s HSD test) tested for autoclaved (n = 3) and non-autoclaved soil (n = 4). No significant differences were observed between the three soil types (non-autoclaved soil, n = 4).

### Breakdown of pure allyl-GLS in soils and effects on the bacterial community composition

As there is little information on whether the soil type can influence degradation of intact GLS and formation of hydrolysis products, both breakdown with and without addition of plant myrosinase was studied in the three soils. Allyl-GLS was used as a model GLS. Moreover, the effect of the treatments on the bacterial community composition was studied in order to evaluate the community response to the treatments.

### Degradation of GLS and formation of breakdown products

**Fig 2:**
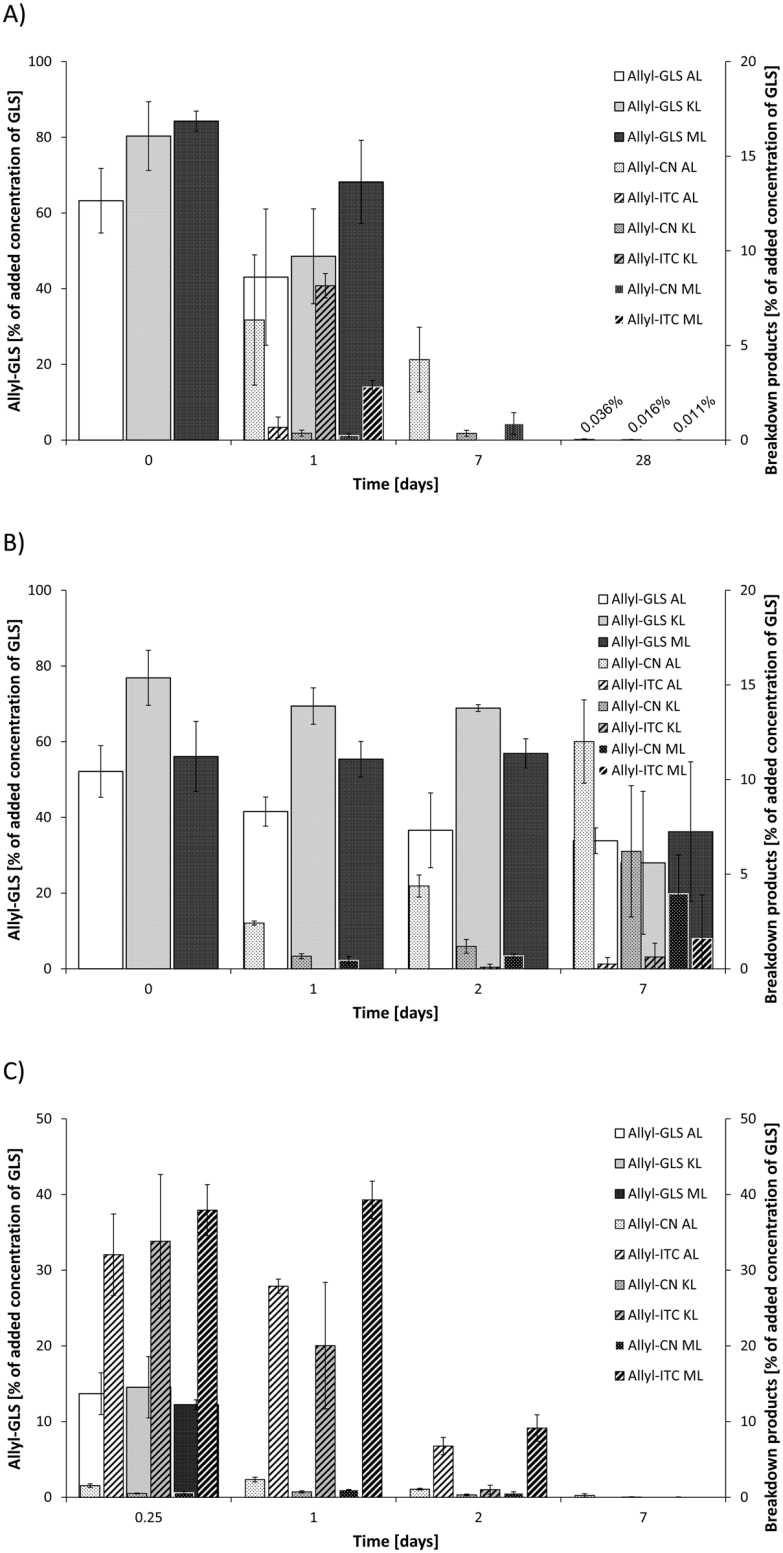
Breakdown of allyl-GLS in three different soils (AL, KL, and ML). A) Degradation of pure allyl-GLS (0.32 μmol/g soil) in soil (treatment I). B) Degradation of pure allyl-GLS (0.32 μmol/g soil) in autoclaved soil (treatment I). C) Degradation of pure allyl-GLS (0.16 μmol/g soil) in soil with addition of 0.1 U/g myrosinase (treatment II). GLS: glucosinolate; allyl-CN: 3-butenylnitrile; ITC: isothiocyanate. Given are means and standard deviations of three (2B) or four replicates (2A, 2C).

Allyl-GLS (0.32 μmol/g soil) was incorporated into the three different soils in order to study its stability and breakdown behavior with and without the addition of plant myrosinase (treatment I and II, respectively). The results are presented in Fig 2.

When applying allyl-GLS to soil without addition of plant myrosinase, samples were taken immediately after mixing to analyze the initial concentration. The initial recovery of the added allyl-GLS in the AL soil was observed to be lower compared to KL or ML soil (Fig 2A). The concentration of the allyl-GLS declined quickly in all soils, and after seven days no GLS could be detected in the soils.

With regard to the volatile degradation products allyl-ITC and allyl-CN were identified as the main breakdown products. Surprisingly, their formation differed widely: whereas allyl-ITC was the main product in KL and ML soil (with 26% and 17% recovery of the degraded allyl-GLS), in the AL soil the nitrile was formed as the main degradation product (Fig 2A). 31% of the degraded allyl-GLS was recovered as the allyl-CN in AL soil, but only 3% as the corresponding ITC. In the other soils only 1–2% of degraded GLS was recovered as the nitrile but 17–26% as the ITC. Moreover, whereas the ITC quickly declined too, the nitrile was more persistent in the soil and could be detected in all soils even after four weeks (Fig 2A). As iron can induce nitrile-formation both enzymatically as well as by a chemical degradation pathway [1,42], the soluble iron content of the three different soils was analyzed by AAS. The AL soil contained 2.8 times as much soluble iron as the ML and KL soils (Table 1). Moreover, a low pH value is known to favor nitrile formation during enzymatic degradation [43]. Measurement of the pH value of the soils revealed that pH value of AL soil was slightly lower than in the other two soils (Table 1).

In our study pure allyl-GLS was quickly degraded in all soils. Previous studies with pure GLS made extracellular, probably not plant-derived myrosinase (no Brassicales plants grown for at last five years) responsible for GLS degradation, as benzyl-GLS degradation was stopped in autoclaved soils but not in soils treated by γ-irradiation or azide, that do not inactivate enzymes [26]. Therefore, here we also studied the degradation of allyl-GLS in autoclaved soil to reveal whether non-enzymatic degradation will occur. As shown in Fig 2B, the GLS was much more stable compared to native soil (Fig 2A). It was observed that all three autoclaved soils formed the allyl-CN as the major degradation product, which was already present after 24 h of incubation. Again, this compound was particularly formed in the AL soil and made up to 23% of the degraded allyl-GLS after 24 h. After seven days the allyl-ITC was also present, its concentrations being highest in the ML soil. In this soil it represented 8% of the degraded GLS, whereas the allyl-CN represented 16% (Fig 2B). In another experiment we studied enzymatically induced degradation of allyl-GLS (0.16 μmol/g soil) and formation of breakdown products in native soil by adding plant myrosinase (treatment II). The results are presented in Fig 2C. The allyl-GLS was quickly hydrolyzed by treatment II and after 24 h it was completely degraded in all soils. After 6 h of incubation in all soils similar allyl-ITC concentrations were found (32–38% of initial GLS concentration being 0.054–0.060 μmol/g soil, respectively) (Fig 2C). After 24 h the ITC concentrations were higher than those observed during treatment I (compare Fig 2A and 2C). During treatment II the nitrile was also formed in minor concentrations. Again, its concentrations were highest in the AL soil. The decline of the formed allyl-ITC differed among the soils (Fig 2C).

### Influence of pure allyl-GLS breakdown on microbial community

**Fig 3:**
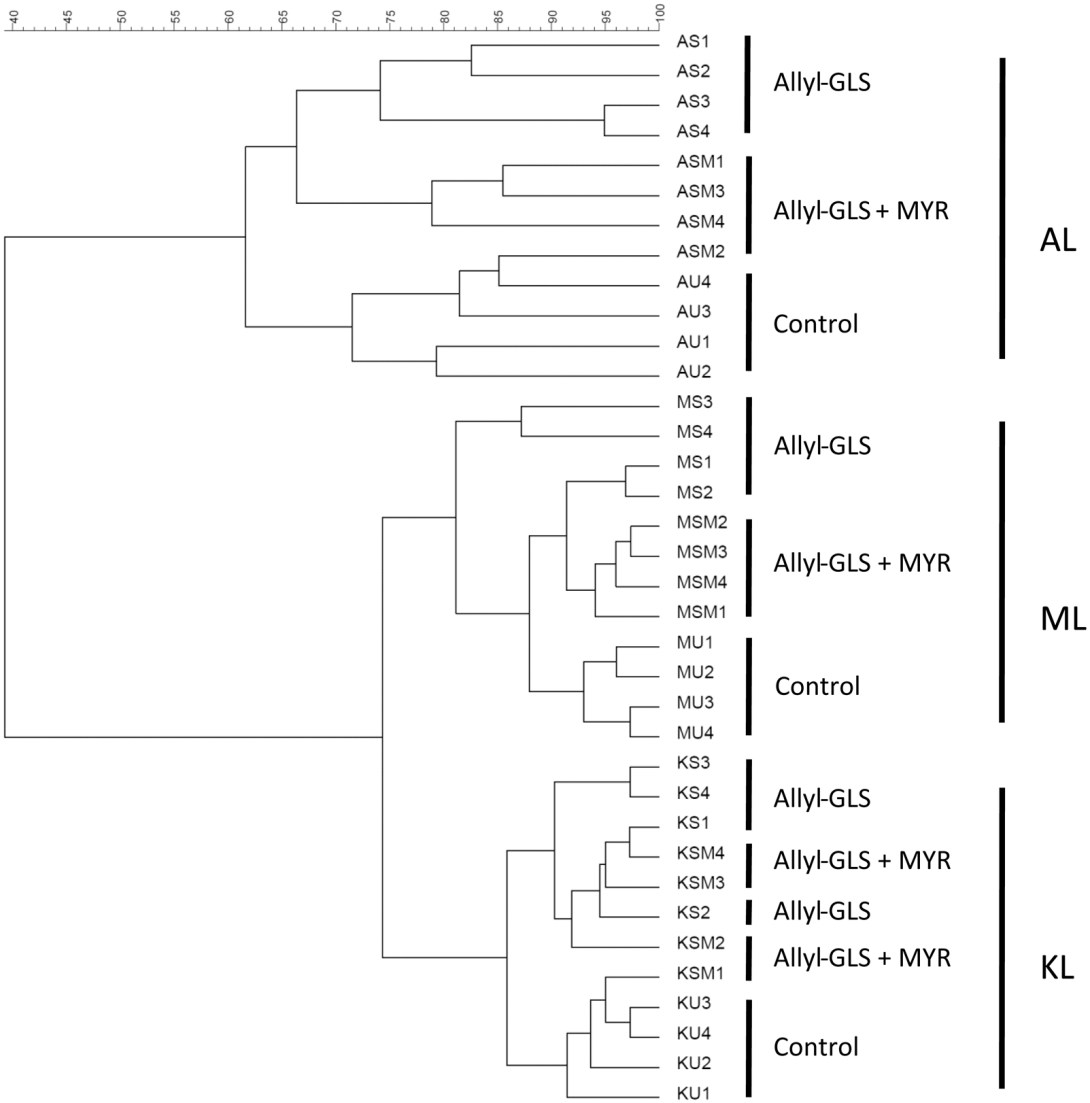
Cluster analysis of bacterial communities from soils as the effect of allyl-GLS and allyl-GLS + myrosinase (MYR) seven days after treatment. Displayed are all four replicates (1–4), Gel-Compar II 6.5. Soil treated in a similar way but without the addition of GLS-treatment was used as a control. The first letter (A, M, or K) indicates the soil, the second letter(s) indicate(s) the treatment (S: sinigrin = allyl-GLS; SM: sinigrin + MYR; U: untreated = control), and the numbers 1–4 indicate the repetitions.

In order to evaluate the effects of the soil treatment with pure allyl-GLS with or without myrosinase on soil bacterial communities (treatments I and II), samples taken after seven days of incubation at room temperature were subjected to total community (TC-) DNA extraction and DGGE analysis of 16S rRNA gene fragments amplified from TC-DNA. The UPGMA analysis based on Pearson correlation coefficient for each pair of lanes grouped the fingerprints according to their similarity using the hierarchical cluster method which is presented in Fig 3.

In general, a separation of the cluster from the control soil and the soil treated with both pure allyl-GLS with or without myrosinase was observed in all soils (AL, KL, and ML). The cluster analysis also revealed soil type-dependent clustering in which ML and KL soils were more similar to each other, sharing around 75% similarity. In contrast, the AL soil was distinct from the other two soils, and shared a low similarity of around 40% with ML and KL soil (Fig 3). Overall, each soil type presented individual clustering which showed significant dissimilarities to each other of 30.32%. Within soil type, the treatments showed small but significant effects on the bacterial community composition in the soils which is indicated by the dissimilarity (d)-values of 14.29%, 6.17% and 6.36% for AL, ML, and KL soils, respectively. Significant changes of bacterial communities were also observed between the soil treated with allyl-GLS and the soil treated with allyl-GLS combined with myrosinase (Table 3). Interestingly, higher d-values for the comparison of control to soil treated with allyl-GLS than for the comparison of control to allyl-GLS with myrosinase point to a more pronounced effect of the pure allyl-GLS on the bacterial community composition.

**Table 3:** Pairwise comparisons between bacterial similarity measures obtained within soil replicates.

Soil type and treatment	Dissimilarities (%)
AL	
Control vs. allyl-GLS	21.95*
Control vs. allyl-GLS + MYR	9.94*
Allyl-GLS vs. allyl-GLS + MYR	10.98*
KL	
Control vs. allyl-GLS	10.90*
Control vs. allyl-GLS + MYR	4.40*
Allyl-GLS vs. allyl-GLS + MYR	3.80*
ML	
Control vs. allyl-GLS	9.58*
Control vs. allyl-GLS + MYR	4.80*
Allyl-GLS vs. allyl-GLS + MYR	4.13*

Average similarity of fingerprints between treatments,

* indicates significant differences between treatments,

P < 0.05

## Discussion

In the present study the fate of *Brassica* plant derived GLS hydrolysis products, pure allyl-ITC, as well as the breakdown of pure allyl-GLS and the related effects on bacterial community have been studied. The effects were investigated using three reference soils from Rosaceae rootstock nurseries affected by replant disease. With regard to the first experiment, that investigated the breakdown of different plant-derived hydrolysis products, upon tissue homogenization the Indian mustard was observed to release allyl-ITC and 2-phenylethyl-ITC as the main degradation products from the GLS. This is in accordance with the literature [11,12]. As radishes are known for their high content of 4-MT-3But GLS with its ITC being the main hydrolysis product [22,44], the observed formation of the 4-MT-3But-ITC from the oilseed radish agrees with previous findings, too.

In the soils the plant-derived GLS hydrolysis products as well as pure allyl-ITC were not stable but their concentrations declined rapidly. Decline of ITC concentration can be caused by different effects: next to volatilization of volatile ITC such as allyl-ITC, they readily react with nucleophiles such as hydroxyl ions, amines or thiols [21] and thus can be bound to soil matrix or can be degraded by soil microbiota [16].

In the present study the soil types did not affect the degradation of GLS breakdown products. Factors such as the pH value surely would influence ITC stability [21], but as all three soils had all slightly acidic pH values and were relatively similar in soil type (sandy soils) (Table 1) probably no differences occurred. In the study of Gimsing et al. the degradation of benzyl-ITC did not differ between the A horizons of two soils (pH values of 6.9 and 7.2) but was increased in subsoils (pH 6.1 and 7.6) [25]. Moreover, the half-life periods of benzyl-ITC during (storage at 8°C, 13–14% water content) reported for the A-horizon (34.6 h and 40.3 h) [25] were similar to the results obtained for 2-phenylethyl-ITC in the present study with a soil: water ratio of 1: 0.5 (S1 Table). Storage of ITC at 25°C in the same A-horizon soil with much higher water content (soil: water 1:1) resulted in much lower half-life periods (0.9–1.9 h) in the same A-horizon soils [18], indicating that ITC are more stable in soils with low water content.

With regard to the stability of individual hydrolysis products great differences were observed in the present study: oilseed radish derived 4-MTB-ITC was the least stable of all hydrolysis products with a half-life of about only one hour (S1 Table and Fig 1). The chemically similar 4-MT-3But-ITC was also very labile while alk(en)yl-ITC, CETP, and aromatic ITC products released from Indian mustard were much more stable (S1 Table, Fig 1). It is unlikely that the differences in concentration between the various hydrolysis products were caused by a shift in pH induced by the plant material, as the freshly homogenized oilseed radish and Indian mustard tissue had the same pH value (pH 6.0). The rapid decline in the soils of these methylthioalkyl ITC probably is based on their instability caused by the double bond in α-position to the sulfanyl group [1] as 4-MT-3But-ITC was shown to be very reactive and to form a variety of breakdown and addition products in aqueous media [45,46]. The oxidized analog was shown to be very labile as well [47].

Stability of nitriles usually was much higher compared to their ITC analogs. The relatively high chemical stability of aliphatic and aromatic nitriles was also demonstrated previously [32]. The 4-MT-3But-CN present in very low concentrations after hydrolysis of oilseed radish GLS seems to be unstable as well as its analogous ITC, probably due to the corresponding unsaturated structure.

Next to the chemical structure, a major influence on ITC stability seems to be the microbial community in the soil, as autoclaving significantly increased the stability of pure allyl-ITC. However, in contrast to Gimsing et al. [18], the ITC decline was still observable and had become significant already after 48 h. This might be caused due to re-colonization of the soil samples by microorganisms, since the incubation did not take place under sterile conditions.

In the second part of the present study, degradation of pure allyl-GLS was studied with or without addition of myrosinase, and the treatment effects on the bacterial community were investigated as well. When pure allyl-GLS was added to the soils the initial recovery was below 100% and was lower in the AL soil compared to KL or ML soil (Fig 2A). This loss probably is due to GLS adsorption to soil compounds. Humic acid but also minerals such as iron containing goethite were shown to adsorb GLS to the surface, the effect increasing at acidic pH values resulting in a rapid decline in extractable GLS [48]. In the present experiment, the GLS concentration quickly declined. The decline is in accordance with the results of other groups who reported half-lives of GLS ranging from 3.5 to 15.5 h at 20°C differing between the GLS, soil type and increasing with the water content of the soil [26,30]. Due to pure allyl-GLS degradation in the present study ITC but also nitriles were identified to be the main degradation products.

To our knowledge, this is the first report on nitrile being a major degradation product of pure GLS in soil. In the study by Gimsing et al. the nitrile was detected in the subsoil (B-horizon) after degradation of pure benzyl-GLS. However, its concentration was very low in that study [25]. Probably, the formation of the nitrile as the major breakdown product from allyl-GLS degradation in the present study was due to the relatively high content of iron and maybe also to the slightly lower pH value of the AL soil compared to the two other soils. However, as differences between the pH-values were small, presumably mainly the iron content of the soil influenced allyl-GLS degradation. Autoclaving the soil resulted in stabilizing the GLS as was previously reported [26]. Autoclaving does not only inactivate microorganisms that might be able to degrade GLS but also myrosinase. Although no Brassicales plants have been grown on the soils for more than five years, it cannot be ruled out that the degrading effect in non-autoclaved soil is caused by presence of extracellular myrosinase [26]. Probably, as a multitude of microorganisms have been shown to be able to degrade GLS, microbial myrosinases are mainly responsible for the degradation of GLS. In contrast to the literature [26] the slower degradation after autoclaving resulted still in the formation of nitriles that were the major breakdown products in all three soils (Fig 2C). It has been observed that autoclaving of soil can increase concentration of ferrous ions [49], supporting the hypothesis that iron (II) ions affect the fate of GLS during degradation. Therefore, this nitrile formation seems to be mainly chemically induced, probably by a non-enzymatic, iron (II)-induced mechanism reported previously [42]. Moreover, the present study indicates that the nitriles are the main non-enzymatically induced degradation products of GLS in soils. When myrosinase as well as allyl-GLS were added to the soil, the GLS was quickly converted into the corresponding ITC. Surprisingly, the decline of the formed allyl-ITC in the further course of the experiment differed within the three soils. This is in contrast to the experiment with pure allyl-ITC, which revealed no differences within the soils (compare Fig 2C and S1 Table). Possibly the presence of the GLS somehow promoted microbes able to degrade the ITC especially in the KL soil.

Studying the effects of treatments I (addition of allyl-GLS without myrosinase) and II (with myrosinase) on the bacterial community composition of the soils revealed significant dissimilarities between the three soil types. The soil type-dependent clustering of the DGGE fingerprints indicated that the soils harbored different bacterial community composition as observed in many other studies [50–52]. The high similarity clustering of ML and KL soils might be due to the similar cultivation history of the soils. On both soils, rose rootstocks had been grown earlier, and root exudates from the plants probably were able to select similar bacterial communities [53,54]. Moreover, these two soil types also have comparable soil properties, e.g. pH value, organic matter, or C/N content (Table 1). In contrast to ML and KL soils, apple rootstocks had been grown in AL soil for a long time. Then, plum (*Prunus domestica*) and quince (*Cydonia oblanga*) had been grown in 2010 and 2011, respectively. Moreover, AL soil had a higher iron content and a slightly lower pH value, thereby probably promoting a different bacterial community. After GLS addition to the soil, the treatments led to significant changes of bacterial communities in all soil types, which is in agreement with the findings of Hu et al.: Depending on incubation time and the GLS breakdown product, the bacterial composition in phylum *Firmicutes*, genera *Lysinibacillus* and *Paenibacillus*; *Bacteroidetes* and *Acidobacteria* were shown to be pronouncedly affected [55].

In the present study, surprisingly, the application of allyl-GLS without myrosinase addition showed stronger effects on the bacterial communities in the soil than the combination of allyl-GLS with myrosinase (compare d-values in Table 3). Moreover, the effect of allyl-GLS was most distinctive in the AL soil and least in ML. As discussed above allyl-GLS in AL soil was degraded mainly to nitrile. This suggests that ITC-formation is not responsible for the observed effects as allyl-ITC concentration was highest after treatment II (with myrosinase). Several bacteria are able to hydrolyze GLS and probably feed on the released glucose [56]. Moreover, microbial myrosinases were shown to increase when adding GLS to soil [30]. In the study of Omirou et al. [30] pure 4-(methylthio)butyl- and 4-(methylsulfinyl)butyl GLS increased soil microbial respiration rate but did not change microbial biomass carbon. Therefore, it is suspected that several bacteria can proliferate due to the addition of GLS. Those bacteria that can degrade GLS to the less toxic nitrile probably will grow faster, which might explain the pronounced effect of allyl-GLS treatment on the bacterial community in the AL soil.

## Conclusions

The results of the present study clearly show that the stability of GLS hydrolysis products in soil is mainly influenced by microbiota present in the soil but also by their chemical structure, as methylthioalk(en)yl hydrolysis products were least stable. Usually nitriles were more stable compared to the corresponding ITC. With regard to the degradation of pure allyl-GLS, the formation of its degradation products (nitrile or ITC) was shown to be dependent on the soil type. Iron content of the soil was assumed to be a major factor favoring nitrile formation in sandy soils with acidic pH value. Allyl-GLS without myrosinase addition had a significantly higher impact on the bacterial communities within the soils than the treatment with allyl-GLS with myrosinase. Thus GLS probably will alter bacterial communities by an increased relative abundance of bacteria able to use GLS as an additional C-source as well as the decrease in abundance of bacterial populations impacted by the degradation products. Addition of intact GLS therefore might have greater effects on soil microbial communities than biofumigation practices with release of ITC. Identification of the bacterial strains that are affected by GLS and their functional effects in the soil and on plant growth will be the focus of future studies in this context.

## Supporting Information

S1 Table Kinetic parameters obtained by fitting of the eqs 1 and 2. Means and standard deviations of three replicates are given. Small letters in brackets indicate significant differences between the half-life periods of the different GLS hydrolysis products at the p < 0.05 level (Fischer LSD test) tested for each soil individually. ^a^ITC: isothiocyanate; ^b^CETP: 1-cyano-2,3-epithiopropane, ^c^4-MTB-ITC: 4-(methylthio)butyl ITC; ^d^4-MT-3But-ITC: 4-(methylthio)-3-butenyl ITC.
(DOCX)
