# Supplementary material for: Degradation of Biofumigant Isothiocyanates and Allyl Glucosinolate in Soil and Their Effects on the Microbial Community Composition
Source: PLoS One. 2015 Jul 17;10(7):e0132931. doi: 10.1371/journal.pone.0132931 (PMC4505889; doi:10.1371/journal.pone.0132931)
Supplement: S1 Table — Means and standard deviations of three replicates are given. Small letters in brackets indicate significant differences between the half-life periods of the different GLS hydrolysis products at the p < 0.05 level (Fischer LSD test) tested for each soil individually. aITC: isothiocyanate; bCETP: 1-cyano-2,3-epithiopropane, c4-MTB-ITC: 4-(methylthio)butyl ITC; d4-MT-3But-ITC: 4-(methylthio)-3-butenyl ITC. (DOCX) [file pone.0132931.s001.docx]

**S1 Table: Kinetic parameters obtained by fitting of the equations 1 and 2.**

|  | GLS hydrolysis product | Soil | k [h^-1^] | t_1/2_ [h] | R^2^ |
| --- | --- | --- | --- | --- | --- |

| Indian mustard | Allyl-ITC^a^ | AL | 0.037 ± 0.006 | 19.07 ± 3.13 (ab) | 0.91 ± 0.01 |
| --- | --- | --- | --- | --- | --- |
|  |  | KL | 0.037 ± 0.011 | 19.78 ± 6.25 (ab) | 0.96 ± 0.04 |
|  |  | ML | 0.051 ± 0.015 | 14.54 ± 4.42 (ab) | 0.95 ± 0.04 |
|  |  |  |  |  |  |
|  | CETP^b^ | AL | 0.032 ± 0.012 | 23.60 ± 8.06 (ab) | 0.84 ± 0.12 |
|  |  | KL | 0.015 ± 0.006 | 51.12 ± 23.02 (cd) | 0.68 ± 0.16 |
|  |  | ML | 0.031 ± 0.008 | 22.89 ± 4.82 (bc) | 0.78 ± 0.09 |
|  |  |  |  |  |  |
|  | sec-Butyl-ITC | AL | 0.022 ± 0.009 | 35.10 ± 16.04 (b) | 0.63 ± 0.17 |
|  |  | KL | 0.013 ± 0.002 | 52.91 ± 8.90 (d) | 0.76 ± 0.27 |
|  |  | ML | 0.030 ± 0.016 | 26.60 ± 11.20 (bc) | 0.68 ± 0.13 |
|  |  |  |  |  |  |
|  | 2-Phenylethyl-ITC | AL | 0.033 ± 0.029 | 41.32 ± 40.06 (b) | 0.72 ±0.23 |
|  |  | KL | 0.025 ± 0.011 | 31.69 ± 12.35 (bc) | 0.79 ± 0.33 |
|  |  | ML | 0.026 ± 0.012 | 31.31 ± 16.14 (c) | 0.61 ± 0.20 |
|  |  |  |  |  |  |
| Oilseed radish | 4-MTB-ITC^c^ | AL | 0.586 ± 0.037 | 1.19 ± 0.08 (a) | 1.00 ± 0.00 |
|  |  | KL | 0.603 ± 0.052 | 1.16 ± 0.10 (a) | 0.99 ± 0.01 |
|  |  | ML | 0.575 ± 0.080 | 1.22 ± 0.17 (a) | 0.99 ± 0.01 |
|  |  |  |  |  |  |
|  | 4-MT-3But-ITC^d^ | AL | 0.238 ± 0.007 | 2.91 ± 0.08 (a) | 0.96 ± 0.02 |
|  |  | KL | 0.223 ± 0.017 | 3.11 ± 0.23 (a) | 0.97 ± 0.01 |
|  |  | ML | 0.219 ± 0.010 | 3.16 ± 0.14 (a) | 0.96 ± 0.04 |

Means and standard deviations of three replicates are given. Small letters in brackets indicate significant differences between the half-life periods of the different GLS hydrolysis products at the p < 0.05 level (Fischer LSD test) tested for each soil individually. ^a^ITC: isothiocyanate; ^b^CETP: 1-cyano-2,3-epithiopropane, ^c^4-MTB-ITC: 4-(methylthio)butyl ITC; ^d^4-MT-3But-ITC: 4-(methylthio)-3-butenyl ITC
